# Variables associated with unplanned general adult ICU admission in hospitalised patients: protocol for a systematic review

**DOI:** 10.1186/s13643-017-0456-0

**Published:** 2017-03-28

**Authors:** James Malycha, Tim Bonnici, Katarina Sebekova, Tatjana Petrinic, Duncan Young, Peter Watkinson

**Affiliations:** 1 Kadoorie Centre for Critical Care Research and Education, John Radcliffe Hospital, Level 3, Headley Way, Oxford, OX3 9DU UK; 2 University of Oxford, Bodleian Health Care Libraries, Academic Centre, John Radcliffe Hospital, Level 3, Headley Way, Oxford, OX3 9DU UK

## Abstract

**Background:**

Failure to promptly identify deterioration in hospitalised patients is associated with delayed admission to intensive care units (ICUs) and poor outcomes. Existing vital sign-based Early Warning Score (EWS) algorithms do not have a sufficiently high positive predictive value to be used for automated activation of an ICU outreach team. Incorporating additional patient data might improve the predictive power of EWS algorithms; however, it is currently not known which patient data (or variables) are most predictive of ICU admission. We describe the protocol for a systematic review of variables associated with ICU admission.

**Methods/design:**

MEDLINE, EMBASE, CINAHL and the Cochrane Library, including Cochrane Database of Systematic Reviews and the Cochrane Central Register of Controlled Trials (CENTRAL) will be searched for studies that assess the association of routinely recorded variables associated with subsequent unplanned ICU admission. Only studies involving adult patients admitted to general ICUs will be included. We will extract data relating to the statistical association between ICU admission and predictor variables, the quality of the studies and the generalisability of the findings.

**Discussion:**

The results of this review will aid the development of future models which predict the risk of unplanned ICU admission.

**Systematic review registration:**

PROSPERO: CRD42015029617

**Electronic supplementary material:**

The online version of this article (doi:10.1186/s13643-017-0456-0) contains supplementary material, which is available to authorized users.

## Background

Unplanned admission of a patient in an acute care hospital to an intensive care unit (ICU) is a frequent occurrence [1]. Analysis of the Intensive Care National Audit and Research Centre (ICNARC) database shows that in 2012 roughly 40,000 patients had an unplanned ICU admission with up to 80% of these patients experiencing a preceding period of unchecked clinical deterioration [2–4]. Their mortality rate was 1.5 times that of patients admitted directly to ICU from the emergency department (30.3% versus 19.7%). Timely admission to an ICU may improve outcomes for these patients [5]. Many institutions worldwide use risk scores to trigger escalation in care. Escalation of care based on an Early Warning Score (EWS) is mandated in the UK [6].

Despite implementation of EWS systems, missed clinical deterioration remains a significant problem [7]. Cognitive errors and barriers to communication have been identified as causes of missed deterioration [8]. In an attempt to bypass these problems, some institutions have trialled directly linking electronic vital sign charts to alerting systems [9]. However, existing EWS algorithms, which are typically based on vital signs, have a poor positive predictive value for severe deterioration [10]. Therefore, they cannot be usefully deployed in systems which aim to automatically alert trained specialists to impending deterioration on the ward as the number of false alerts is excessive. Inclusion of additional variables can improve the accuracy of EWS models [11, 12].

### Objective

We will conduct a systematic review to identify studies of patient-derived variables that are associated with an increased risk of unplanned ICU admission. For the purposes of the review, a variable is defined as an indivisible entity, as opposed to a composite entity such as a risk score, which is made up of multiple variables. A patient-derived variable is a measure of the properties of a patient as opposed to a measure of institutional processes such as nurse-to-patient ratio or number of escalation calls.

## Methods/design

This protocol will adhere to the requirements of Preferred Reporting Items for Systematic Reviews and Meta-analyses Protocol (PRISMA-P), which is included as Additional file 1.

### Search strategy

Papers will be identified by searching Medical Literature Analysis and Retrieval System Online (MEDLINE), Excerpta Medica database (EMBASE), Cumulative Index to Nursing and Allied Health Literature (CINAHL), the Cochrane Database of Systematic Reviews and the Cochrane Central Register of Controlled Trials (CENTRAL). We will include additional papers from the references of reviews articles or studies identified during screening and papers from the authors’ personal libraries. A full description of the search strategy is outlined in Appendix 1.

### Study selection

Two researchers will independently screen titles and abstracts of identified papers against the inclusion and exclusion criteria. They will not be blinded to the journal titles or to the study authors or institutions. If there is disagreement or uncertainty regarding eligibility, the article will be included in the next stage of screening for further analysis for inclusion/exclusion. The full text will be retrieved for all articles not excluded by the initial screening. These papers will be independently assessed against the inclusion and exclusion criteria. Disagreements about eligibility will be resolved by discussion between the screening researchers or a third party.

### Inclusion criteria

#### Types of studies

Quantitative studies published in peer reviewed journals assessing adults admitted to adult hospitals will be eligible for inclusion in this review. Studies will most likely be prospective or retrospective cohort and case-control studies.

#### Study characteristics

Eligible studies must include both a cohort of patients admitted to ICU and a cohort not admitted to ICU. Unplanned ICU admission may be either a primary or secondary outcome measure. Studies published from January 2000 until the day of search completion will be included to ensure modern day applicability. No language restrictions will be applied.

#### Phenomenon of interest

Studies must describe a statistical relationship between a patient-derived variable (e.g. heart rate or creatinine level) and an unplanned admission to intensive care from a general ward or emergency department. ‘Diagnosis’ or ‘groups of diagnoses’ are eligible to be included as variables. If a paper analyses both eligible variables (e.g. variables that are widely available in most UK hospitals) and non-eligible variables (e.g. variables that are not widely available in most UK hospitals), it will still be eligible for inclusion, with the authors using only the eligible variables for inclusion in the review.

#### Population

Studies that sample adult patients with an unplanned admission to ICU will be considered for inclusion. For the purpose of this review, adult is defined as >16 years of age. There will be no other restrictions.

### Exclusion criteria

#### Types of studies

Qualitative studies, case studies, grey-literature, editorials, letters, practice guidelines and abstract-only reports will be excluded.

#### Study characteristics

Studies of cohorts defined by a single condition or narrow group of conditions (e.g. trauma or sepsis) will be excluded. We will also exclude studies that do not use a control versus intervention group.

#### Phenomenon of interest

Studies of ICU readmission or admission to ICUs dedicated to narrow cohorts of patients will be excluded (e.g. patients admitted to ICU with acute liver failure).

#### Population

Studies of participants under 16 years old will be excluded.

### Data extraction

Two authors will independently extract data from the papers and supplementary material. All uncertainties regarding data extraction will be resolved by discussion amongst the study team. DistillerSR (Evidence Partners, Ottawa, Canada) will be used to manage the data and identify duplicate search results. All screening and data extraction forms will be implemented within DistillerSR. As part of the development of this protocol the study forms have been piloted and a calibration exercise has been undertaken to ensure good inter-rater agreement.

### Quality assessment

Risk of bias will be assessed using a scoring system adapted from two previous systematic reviews, [13, 14] both of which are adapted from the Newcastle-Ottawa Scale (NOS) [15]. The NOS is a scoring system designed to assess the quality of nonrandomised studies in meta-analyses. Using a ‘star’ system, it attributes a score to a paper after assessing the selection of study groups, the comparability of the groups and the ascertainment of either the exposure or outcome of interest for case-control or cohort studies. The scoring system used in this systematic review is outlined in Appendix 2.

### Data synthesis and analysis

We will conduct a qualitative synthesis of results from included studies. This will be presented descriptively in table and text form. We will extract summary comparison data as odds ratios (95% confidence intervals) where possible. Where sufficient original data is presented we will calculate odds ratios. Where insufficient data is presented we will contact the authors.

## Discussion

As hospitals move towards fully digital patient records, increasing amounts of data are being collected in hospital Clinical Information Systems. Researchers have begun using this resource to develop models to predict patient deterioration based upon electronically captured data [16, 17]. These models are reported to perform better than conventional EWS algorithms but their clinical adoption is not widespread.

Commonly, patient deterioration prediction models aim to accurately predict one of cardiac arrest, death or unplanned ICU admission. This systematic review will be the first to bring together the hospitalised patient factors that are known to be associated with subsequent urgent admission to ICU alone. This is a vital step in starting to use this information to identify patients at risk of ICU admission.

The findings from this review will contribute to the construction of an improved model for the prediction of clinical deterioration and unplanned ICU admission in adult patients on general wards. The findings may also be useful for researchers seeking to improve upon existing work in this field.

## Appendix 1

**Table 1:** Draft search strategy for MEDLINE

1.	(ICU* OR “intensive care” OR “critical care”).ab,ti.
2.	INTENSIVE CARE UNITS/
3.	1 OR 2
4.	(admission* OR admitted OR transfer*).ab,ti.
5.	(“risk assessment*” OR “risk factor*” OR “risk stratif*” OR predict* OR “increased risk*” OR trigger* OR score* OR scoring OR “early warning” OR escalat* OR deteriorat* OR triag* OR “vital sign*” OR model* OR validat*).ab,ti.
6.	3 AND 4 AND 5
7.	limit 6 to (humans AND yr = “2000 ­Current”)
8.	(observational OR “case control*” OR retrospective OR cohort* OR “systematic review*”).ab,ti.
9.	OBSERVATIONAL STUDY/
10.	CASE­CONTROL STUDIES/
11.	RETROSPECTIVE STUDIES/
12.	COHORT STUDIES/
13.	RANDOMIZED CONTROLLED TRIAL/
14.	REVIEW/
15.	COMPARATIVE STUDY/
16.	PROSPECTIVE STUDIES/
17.	VALIDATION STUDIES/
18.	8 OR 9 OR 10 OR 11 OR 12 OR 13 OR 14 OR 15 OR 16 OR 17
19.	7 AND 18
20.	(unplanned OR unexpected OR unanticipated OR emergency OR “rapid response”).ab,ti.
21.	19 AND 20

## Appendix 2

**Table 2:** Risk of bias scoring

Participant selection	Score
Cohort studies	
Selected cohort is very representative of the general hospitalised population	2
Selected cohort is somewhat representative of the general hospitalised population	1
Cohort is not representative of the general hospital population or the selection of the group was not described	0
Case-control studies	
Cases and controls drawn from the same population and population is very representative of the general hospitalised population	2
Cases and controls drawn from the same population and population is somewhat representative of the general hospitalised population	1
Cases and controls drawn from different sources or the selection of groups was not described	0
Comparability of groups	
No differences between the groups explicitly reported unless it was one of these variables that was under investigation, or such differences were adjusted for	2
Differences between groups were not recorded	1
Groups differed	0
Size	
>100 participants in each group	2
<100 participants in each group	1
Adjustment for confounding	1
Adjustment made for confounding factors in data analysis	1
No adjustment for cofounders	0

## Additional file

Additional file 1: PRISMA-P (Preferred Reporting Items for Systematic review and Meta-Analysis Protocols) 2015 checklist: recommended items to address in a systematic review protocol. (DOC 97 kb)

## Abbreviations

CENTRAL: The Cochrane Database of Systematic Reviews and the Cochrane Central Register of Controlled Trials
CINAHL: Cumulative Index to Nursing and Allied Health Literature
EMBASE: Excerpta Medica database
EWS: Early Warning Score
ICU: Intensive care unit
MEDLINE: Medical Literature Analysis and Retrieval System Online
NICE: National Institute of Clinical and Healthcare Excellence
